# Quantifying the area-at-risk of myocardial infarction *in-vivo* using arterial spin labeling cardiac magnetic resonance

**DOI:** 10.1038/s41598-017-02544-z

**Published:** 2017-05-23

**Authors:** Rachel K. Dongworth, Adrienne E. Campbell-Washburn, Hector A. Cabrera-Fuentes, Heerajnarain Bulluck, Thomas Roberts, Anthony N. Price, Sauri Hernández-Reséndiz, Roger J. Ordidge, David L. Thomas, Derek M. Yellon, Mark F. Lythgoe, Derek J. Hausenloy

**Affiliations:** 1 The Hatter Cardiovascular Institute, University College London, London, UK; 2 Centre for Advanced Biomedical Imaging, University College London, London, UK; 3 Biochemistry and Biophysics Center, Division of Intramural Research, National Heart, Lung and Blood Institute, National Institutes of Health, Bethesda, USA; 4 Cardiovascular and Metabolic Disorders Program, Duke-NUS Graduate Medical School, Singapore, Singapore; 5 National Heart Research Institute Singapore, National Heart Centre Singapore, Singapore, Singapore; 6 Department of Cardiovascular Biomedicine, National Institute of Cardiology I. Ch, Mexico City, Mexico; 7 Institute of Biochemistry, Medical School, Justus-Liebig-University, Giessen, Germany; 8 Kazan Federal University, Department of Microbiology, Kazan, Russian Federation; 9 Division of Imaging Sciences and Biomedical Engineering, King’s College London, King’s Health Partners, St. Thomas’ Hospital, London, UK; 10 Centre for Neuroscience, University of Melbourne, Melbourne, Australia; 11 Department of Brain Repair and Rehabilitation, UCL Institute of Neurology, University College London, London, United Kingdom; 12 Leonard Wolfson Experimental Neurology Centre, UCL Institute of Neurology, University College London, London, United Kingdom; 13 The National Institute of Health Research University College London Hospitals Biomedical Research Centre, London, UK; 14 Yong Loo Lin School of Medicine, National University Singapore, Singapore, Singapore; 15 Barts Heart Centre, St Bartholomew’s Hospital, London, UK

## Abstract

T_2_-weighted cardiovascular magnetic resonance (T2-CMR) of myocardial edema can quantify the area-at-risk (AAR) following acute myocardial infarction (AMI), and has been used to assess myocardial salvage by new cardioprotective therapies. However, some of these therapies may reduce edema, leading to an underestimation of the AAR by T2-CMR. Here, we investigated arterial spin labeling (ASL) perfusion CMR as a novel approach to quantify the AAR following AMI. Adult B6sv129-mice were subjected to *in vivo* left coronary artery ligation for 30 minutes followed by 72 hours reperfusion. T_2_-mapping was used to quantify the edema-based AAR (% of left ventricle) following ischemic preconditioning (IPC) or cyclosporin-A (CsA) treatment. In control animals, the AAR by T2-mapping corresponded to that delineated by histology. As expected, both IPC and CsA reduced MI size. However, IPC, but not CsA, also reduced myocardial edema leading to an underestimation of the AAR by T_2_-mapping. In contrast, regions of reduced myocardial perfusion delineated by cardiac ASL were able to delineate the AAR when compared to both T2-mapping and histology in control animals, and were not affected by either IPC or CsA. Therefore, ASL perfusion CMR may be an alternative method for quantifying the AAR following AMI, which unlike T2-mapping, is not affected by IPC.

## Introduction

Novel therapies are still needed to reduce myocardial infarct (MI) size and prevent the onset of heart failure in patients presenting with an acute myocardial infarction (AMI)^1, 2^. Myocardial salvage, which controls for the variability in area-at-risk (AAR) between patients, is a more sensitive measure of cardioprotective efficacy than MI size alone, but it requires the quantification of the AAR in AMI patients^3, 4^. In this regard, T_2_-weighted cardiovascular magnetic resonance (CMR) of the heart has been shown to retrospectively quantify the AAR in clinical cardioprotection studies^5–8^. This technique is based on the ability of T_2_-weighted CMR to detect the myocardial edema that results from acute ischemia-reperfusion injury (IRI)^9^. The area of myocardial edema delineated by T_2_-weighted CMR has been shown to correspond to the AAR quantified by classical histological staining methods in animal AMI models^10–13^, and the AAR measured by coronary angiography jeopardy scores^14^, myocardial single-photon emission computed tomography (SPECT) imaging^15^ and positron emission tomography using 18F-fluorodeoxyglucose^16^.

Despite the promising application of T_2_-CMR for quantifying the AAR, the validity of this CMR technique for quantifying the AAR has been questioned^17^. Furthermore, recent clinical studies have found that, in addition to limiting MI size, some cardioprotective interventions (such as ischemic postconditioning^18^ and remote ischemic conditioning^5, 8^) may actually reduce the extent of myocardial edema leading to underestimation of the AAR measured by T_2_-CMR, thereby obviating the use of this technique for measuring myocardial salvage in these settings. Therefore, a more robust method for quantifying the AAR, which is not affected by the cardioprotective intervention under investigation, is required.

In the current study, we perform a multi-parameter *in vivo* assessment of MI pathology using CMR. Late-gadolinium enhancement (LGE), T_2_ mapping and arterial spin labeling (ASL) perfusion mapping CMR were assessed in the presence of two well-known cardioprotective therapies that have consistently been shown to reduce MI size in the pre-clinical setting, namely ischemic preconditioning (IPC) and cyclosporin-A (CsA). We aimed to investigate the accuracy of ASL perfusion CMR mapping against T_2_-mapping CMR and histology for delineating the AAR following interventions by IPC and CsA.

## Results

### Validation of mouse model of myocardial IRI and cardioprotective treatments

Initial comparison of AAR and MI size quantified by *ex vivo* histological staining was used to validate the IRI model and efficacy of cardioprotective interventions used in this study.

#### Myocardial AAR

**Figure 1:**
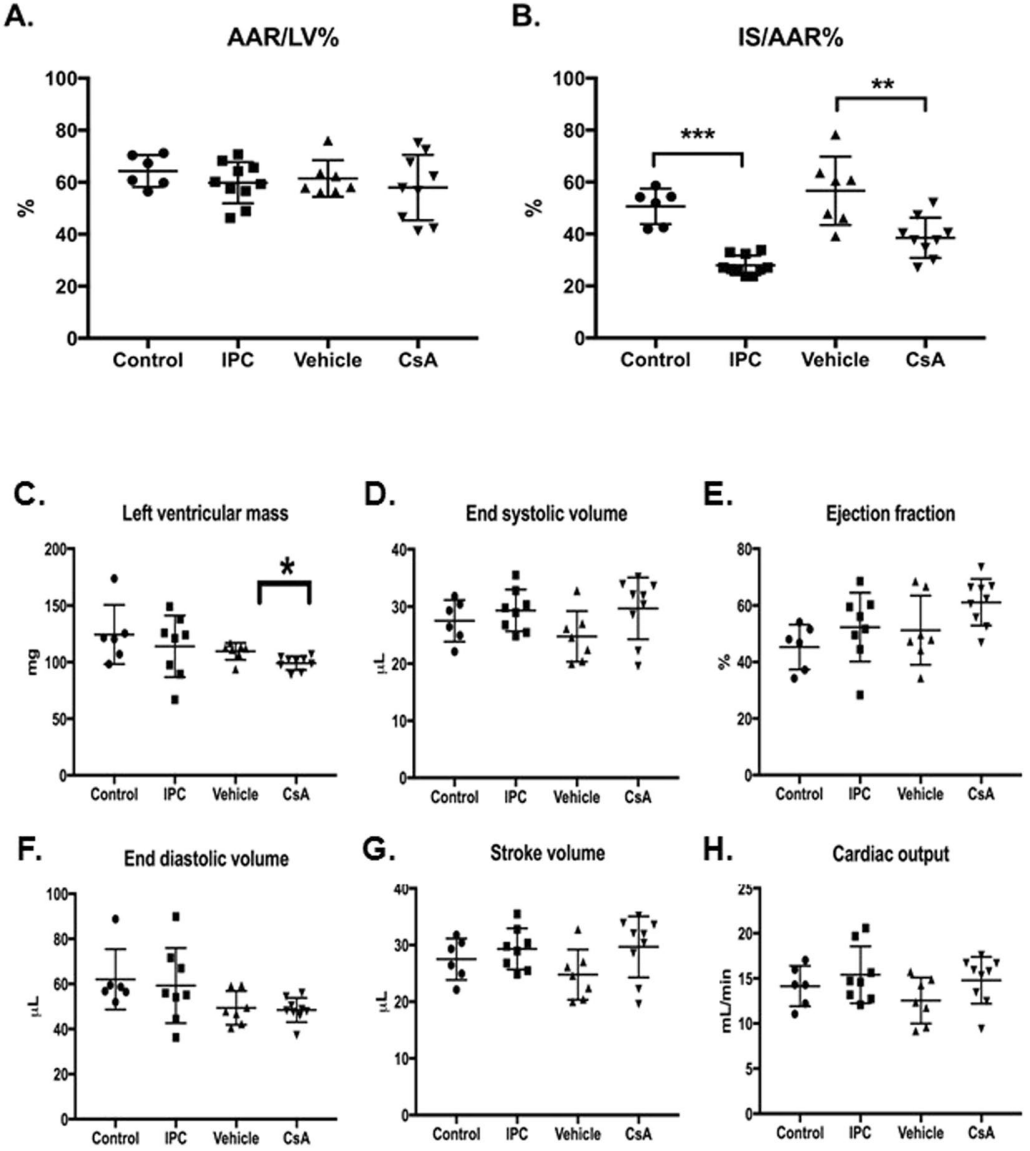
Effect of cardioprotective treatments on area-at-risk, myocardial infarct size in mouse AMI model. (**A**) Myocardial AAR as a fraction of left ventricle area (AAR/LV%) determined by *ex vivo* histological staining. AAR/LV%: control 64.3 ± 6.1, n = 6; IPC 59.8 ± 7.9, n = 10; vehicle 65.4 ± 7.0, n = 8; CsA 58.0 ± 12.6, n = 9; data are mean ± SD; one-way ANOVA p = 0.18. (**B**) Infarct size as a fraction of AAR (IS/AAR%) determined by *ex vivo* histological staining. IPC study: IS/AAR%: control 50.6 ± 6.9, n = 6; IPC 27.9 ± 3.7, n = 10; unpaired t-test ***p < 0.001. CsA study: vehicle 56.6 ± 13.6, n = 7; CsA 38.6 ± 7.8, n = 9; data are mean ± SD; unpaired t-test **p = 0.004. (**C–H)** These figures show the effects of control, IPC, vehicle and CsA on left ventricular mass, end systolic volume, ejection fraction, end diastolic volume, stroke volume and cardiac output. *p = 0.0082.

There was no significant difference in myocardial AAR between treatment groups, determined by *ex vivo* histological staining (Fig. 1A: AAR/LV%: control 64.3 ± 6.1, n = 6; IPC 59.8 ± 7.9, n = 10; vehicle 65.4 ± 7.0, n = 7; CsA 58.0 ± 12.6, n = 9; one-way ANOVA p = 0.62).

#### Cardioprotective efficacy of IPC and CsA treatment protocols

IPC and CsA treatments significantly reduced MI size (infarct size [IS] as a fraction of AAR; IS/AAR%) determined by *ex vivo* histological staining (Fig. 1B: IPC p < 0.0001, CsA p = 0.004). This confirms the effect of cardioprotective treatments for valid evaluation of the CMR protocols examined in this study to quantify putative AAR.

### Cardiac function MRI

The short axis stacks of cine images were analyzed to measure left ventricular mass, end-systolic volume, end-diastolic volume, stroke volume, ejection fraction and cardiac output (Fig. 1C–H). The left ventricular mass was found to be different between vehicle control group and CsA treatment group (p = 0.0082: Fig. 1C). Otherwise, all comparisons were non-significant. Treatment protocols did not appear to modify cardiac function in this acute IRI model.

### Multi-parameteric CMR quantitative summary

**Figure 2:**
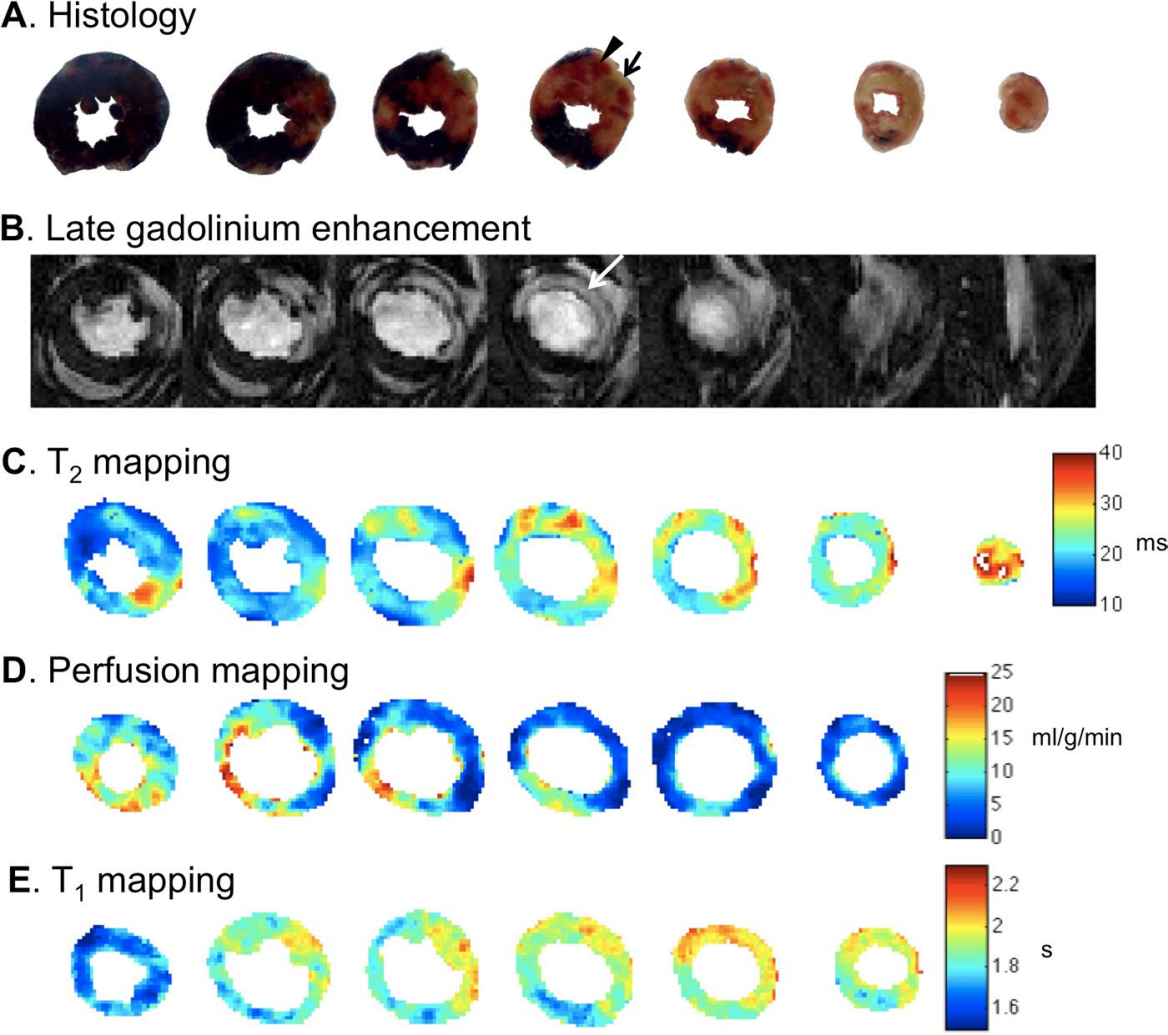
Representative histology and CMR images of a mouse heart subjected to control IRI protocol. (**A**) Histological staining of transverse slices of mouse heart prepared *ex vivo* at approximate positions of MR scanning. TTC staining allows identification of MI as area colored off-white (example indicated by arrow). Evans blue staining allows identification of AAR as area not stained blue (example indicated by arrowhead). (**B**) LGE imaging showing spatially distinct areas of hyper-enhancement in areas of MI (example indicated by arrow). (**C)** T_2_-mapping showing spatially distinct areas of elevated T_2_ (ms) (yellow-red appearance) compared to normal region (dark blue color). (**D**) ASL perfusion mapping showing spatially distinct areas of decreased perfusion (ml/g/min) (dark blue appearance) compared to normal region (yellow-red color). (**E)** T_1_-mapping showing spatially distinct areas of elevated T_1_ (s) (yellow-red appearance) compared to normal region (dark blue color). Histology and MR images have different slice thicknesses and cardiac contraction states and therefore should not be compared slice-by-slice.

MR images and histological staining from an example data set are shown in Fig. 2. The putative IS/LV% calculated by LGE and ASL perfusion mapping (2 SD threshold) are given in Supp. Table 1. The putative AAR/LV% calculated by T_2_ mapping, ASL perfusion mapping (1 SD threshold) and T_1_ mapping are provided in Supp. Table 2. Supp. Table 3 summarizes quantitative T_2_, perfusion and T_1_ values.

### Infarct size as a fraction of the left ventricle area comparison of *in vivo* LGE and *ex vivo* histological staining

Previously validated LGE and histological staining were compared between *in vivo* and *ex vivo* images of transverse myocardial slices.

#### Quantification of MI size in control IRI operated mice

**Figure 3:**
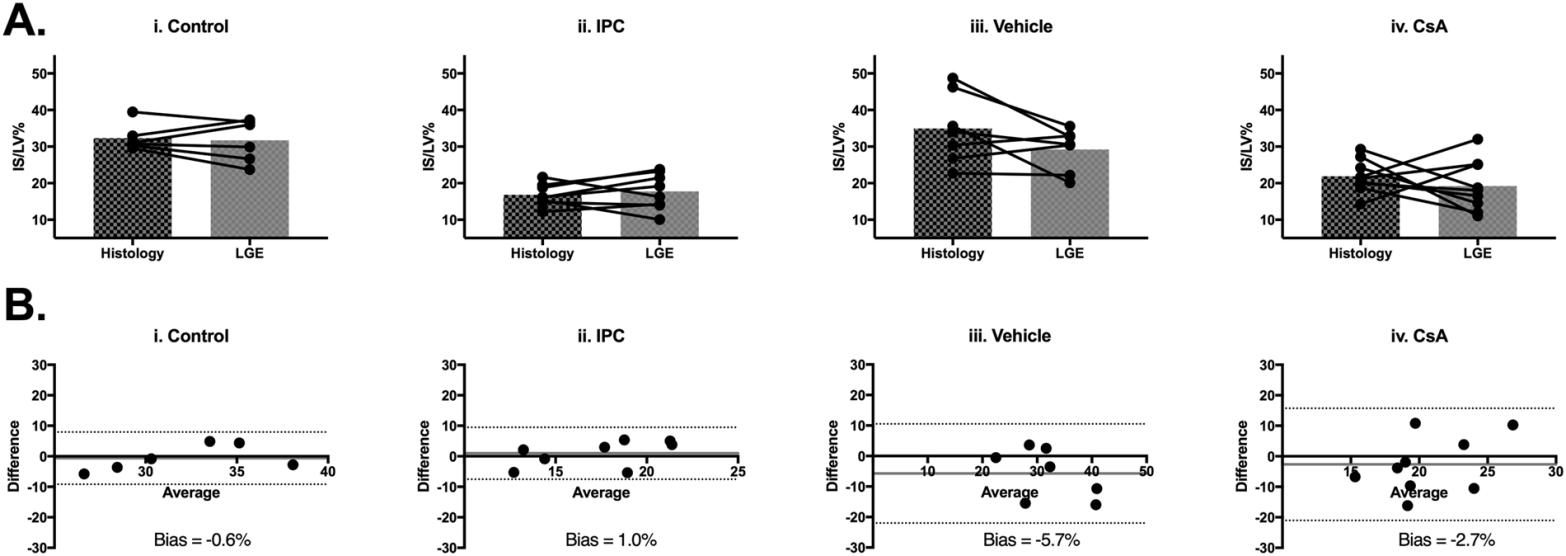
Validation of *in vivo* LGE to determine MI size upon IRI. (**A**) Quantification of infarct size as a fraction of left ventricle area (IS/LV%) by histological staining and LGE for individual animals in control group (n = 6) (i), IPC group (n = 10) (ii), vehicle group (n = 8) (iii) and CsA group (n = 9) (iv). Mean ± standard deviation values are provided in Supp. Table 1. (**B**) Bland-Altman plots comparing histological and LGE methods of estimating IS/LV% for all groups (i–iv).

LGE of mouse hearts subjected to control IRI revealed clearly defined regions of signal enhancement within the left ventricle (Fig. 2B). Quantification of MI size (IS as a fraction of LV area: IS/LV%) in mice subjected to control IRI showed good agreement between LGE and histological staining (Fig. 3Ai: histology IS/LV% 32.3 ± 3.7 versus LGE IS/LV% 31.7 ± 5.8, n = 6, paired t-test p = 0.74).

#### Cardioprotective efficacy of IPC and CsA treatment protocols

LGE of mouse hearts subjected to IRI in the presence of either IPC or CsA, also permitted valid *in vivo* quantification of MI size. There was no significant difference between MI size quantified by *in vivo* LGE and *ex vivo* TTC staining for animals treated with IPC (Fig. 3Aii: histology IS/LV% 16.8 ± 3.0 versus LGE IS/LV% 18.0 ± 4.9, n = 8, paired t-test p = 0.54) or CsA (Fig. 3Aiv: histology IS/LV% 21.9 ± 4.5 versus LGE 19.2 ± 6.9, n = 9, paired t-test p = 0.42). Bland-Altman analysis demonstrated negligible bias in all groups for IS/LV% (Fig. 3B).

### T_2_-mapping provides a valid quantification of myocardial AAR in control IRI mice

**Figure 4:**
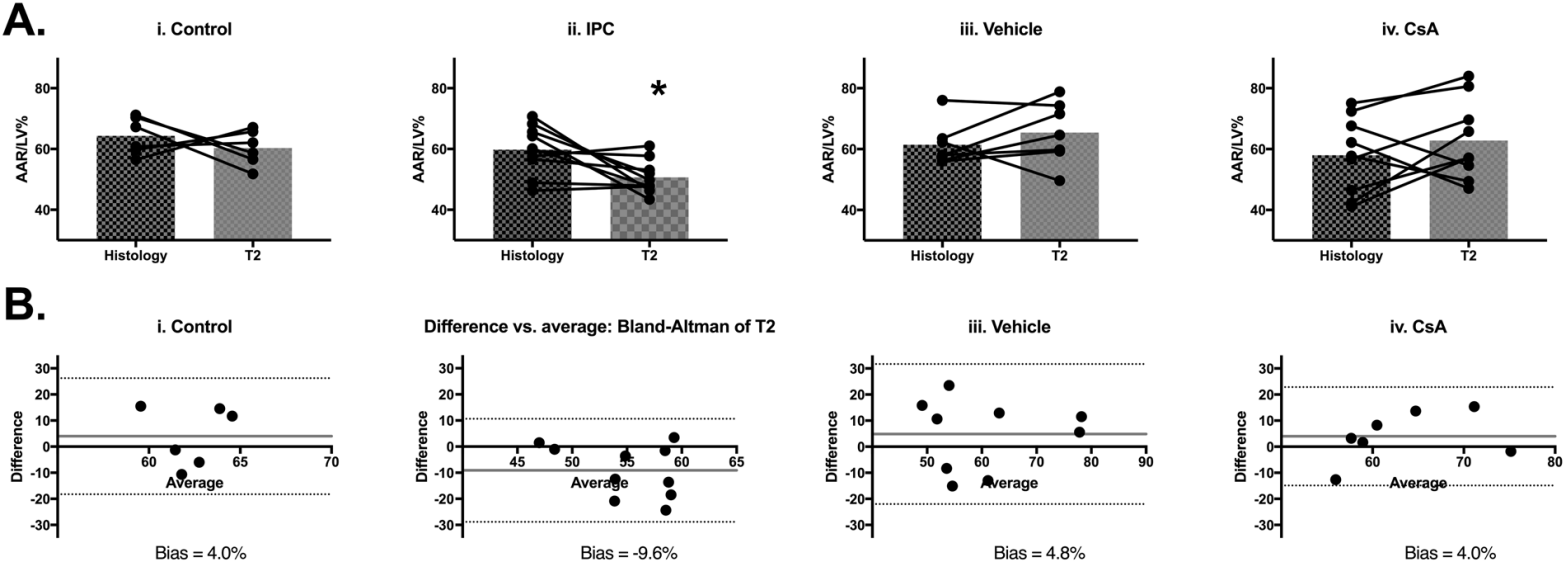
Validation of T_2_ mapping to determine *in vivo* AAR in control and protected hearts upon IRI. (**A)** Quantification of AAR as a fraction of left ventricle area (AAR/LV%) by histological staining and T_2_-mapping animals in control group (n = 6) (i), IPC group (n = 10) (ii), vehicle group (n = 8) (iii) and CsA group (n = 9) (iv). Mean ± standard deviation values are provided in Supp. Table 2. Significant underestimation of AAR/LV% is observed for the IPC group (*p = 0.02). (**B**) Bland-Altman plots comparing histological and T_2_ mapping methods of estimating AAR/LV% for all groups (i–iv).

CMR T_2_-maps of mouse hearts subjected to control IRI revealed clearly defined regions of elevated T_2_-signal within the left ventricle (Fig. 2C). This suggests that this control IRI protocol elicits a detectable change in myocardial water content within the myocardial AAR. Examination of the spatial localization of the regions of elevated T_2_-signal (where T_2_ > mean ROI normal T_2_-signal + 1 standard deviation) was qualified as the putative AAR by comparison with *ex vivo* stained myocardial slices. Quantification of the putative AAR by T_2_-mapping (AAR as a fraction of LV area: AAR/LV%) in mice subjected to control IRI showed good agreement between T_2_-mapping and histological staining (Fig. 4Ai: AAR/LV% histology 64.3 ± 6.1 versus T_2_-mapping 60.3 ± 5.8; n = 6, paired t-test p = 0.43). Bland-Altman analysis provided a small bias of 4.0% (Fig. 4Bi). This confirms that T_2_-mapping may provide a valid method for quantifying myocardial AAR *in vivo* in mice subjected to control IRI.

### Quantification of putative myocardial AAR by T_2_-mapping may be affected by IPC

Application of IPC and CsA was examined in order to determine the validity of *in vivo* myocardial AAR quantification by T_2_-mapping in the presence of these cardioprotective interventions.

#### T_2_-mapping underestimated putative AAR in IPC-treated animals

There was no significant difference in the mean T_2_ values in areas deemed ‘normal’ and ‘elevated’ by threshold analysis of control and IPC treated hearts (Supp. Table 3). However, T_2_-mapping significantly underestimated myocardial AAR in animals treated with IPC (Fig. 4Aii: AAR/LV% histology 59.77 ± 7.9 versus T_2_-mapping 50.7 ± 5.4; n = 10, paired t-test p = 0.04). Bland-Altman analysis provided a bias of −9.1% (Fig. 4Bii). This underestimation of AAR by T_2_ mapping is caused by the reduced edema with the IPC cardioprotective intervention.

#### T_2_-mapping CMR quantification of putative AAR was not affected by CsA treatment

There was no significant difference in the mean T_2_ values in areas deemed ‘normal’ and ‘elevated’ determined by threshold analysis of vehicle and CsA treated hearts (Supp. Table 3). Quantification of putative myocardial AAR by T_2_-mapping was not significantly affected by cardioprotection elicited by administration of CsA (Fig. 4Aiv, AAR/LV% histology 58.0 ± 12.6 versus T_2_-mapping 62.8 ± 13.1; n = 9, paired t-test p = 0.32). Bland-Altman analysis demonstrated a bias of 4.0%, which is equivalent to control and vehicle groups (Fig. 4Biv).

### Perfusion deficit can quantify both putative IS and AAR

ASL perfusion maps of mouse hearts subjected to control IRI revealed clearly defined regions of decreased perfusion within the left ventricle (Fig. 2D). This suggests that this control IRI protocol elicits a detectable change in myocardial perfusion levels after 72 hours myocardial reperfusion. Perfusion was further reduced in the infarct zone and, for the purposes of this study, extreme perfusion deficits (perfusion value < mean ROI perfusion – 2 standard deviations) were qualified as the putative IS by comparison to 6 *ex vivo* myocardial slices. Interestingly, regions of lesser perfusion deficit extended beyond the infarct zone and decreased myocardial perfusion (where perfusion value < mean ROI perfusion – 1 standard deviation) was qualified as the putative AAR for this study. Perfusion deficits were compared with histological analysis of 6 *ex vivo* stained myocardial slices.

**Figure 5:**
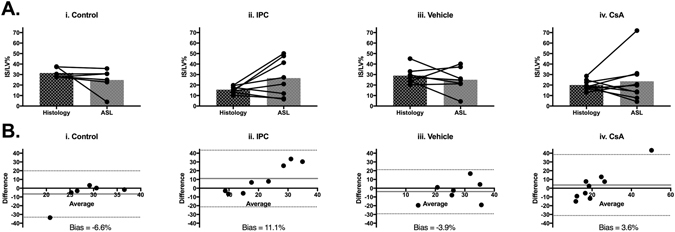
Validation of ASL perfusion mapping to determine *in vivo* MI size using a 2 SD threshold. (**A)** Quantification of MI size as a fraction of left ventricle area (IS/LV%) by histological staining and ASL perfusion mapping with 2 SD threshold for individual animals in animals in control group (n = 6) (i), IPC group (n = 10) (ii), vehicle group (n = 8) (iii) and CsA group (n = 9) (iv). Mean ± standard deviation values are provided in Supp. Table 1. (**B**) Bland-Altman plots comparing histological and ASL perfusion mapping methods of estimating IS/LV% for all groups (i–iv).

Quantification of the putative IS/LV% by ASL perfusion mapping in mice subjected to control IRI using the 2 SD threshold showed good agreement between ASL perfusion mapping and histological staining (Fig. 5Ai: IS/LV% histology 31.5 ± 4.8 versus ASL [2 SD threshold] 24.8 ± 11.2; n = 6, paired t-test p = 0.28). Bland-Altman analysis provided a bias of −6.6% between these two measurements.

**Figure 6:**
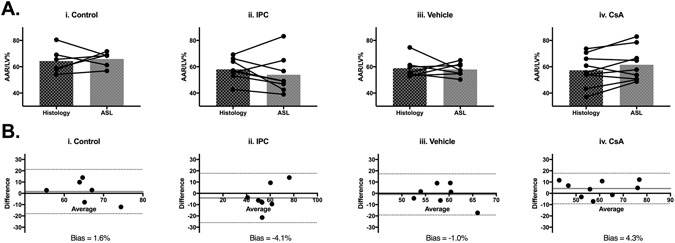
Validation of ASL perfusion mapping to determine *in vivo* AAR size using a 1 SD threshold. (**A**) Quantification of AAR as a fraction of left ventricle area (AAR/LV%) by histological staining and ASL perfusion mapping with 1 SD threshold for individual animals in animals in control group (n = 6) (i), IPC group (n = 10) (ii), vehicle group (n = 8) (iii) and CsA group (n = 9) (iv). Mean ± standard deviation values are provided in Supp. Table 2. (**B**) Bland-Altman plots comparing histological and ASL perfusion mapping methods of estimating AAR/LV% for all groups (i–iv).

Quantification of the putative AAR/LV% by ASL perfusion mapping using the 1 SD threshold showed good agreement between ASL perfusion mapping and histological staining (Fig. 6Ai: IS/LV% histology 64.3 ± 9.6 versus ASL [1 SD threshold] 65.9 ± 5.6; n = 6, paired t-test p = 0.71). Bland-Altman analysis provided a negligible bias of 1.6% between these two measurements. This data, suggests that ASL perfusion mapping may also provide a valid method for quantifying myocardial AAR *in vivo* in mice subjected to control IRI.

### Quantification of putative myocardial IS and AAR by ASL perfusion mapping is not affected by the cardioprotective interventions tested

As described above for T_2_-mapping MR, the effects of IPC and CsA treatment on quantification of the putative AAR quantified by ASL perfusion mapping were examined.

ASL perfusion mapping of putative IS and AAR was not affected by IPC treatment: Using the 1 SD threshold (putative AAR) or 2 SD threshold (putative IS), there was no significant difference in the mean perfusion value in areas deemed ‘normal’ or ‘reduced’ perfusion threshold (Supp. Table 3).

IS/LV% calculated using ASL perfusion mapping [2 SD threshold] was not significantly different from histology (Fig. 5Aii: IS/LV% Histology 15.7 ± 3.3, ASL 26.6 ± 17.9, paired t-test p = 0.28). However, significant variability in this group generated a large bias of 11.0% using Bland-Altman analysis (Fig. 5Bii).

AAR/LV% calculated by ASL perfusion mapping [1 SD threshold] was not significantly affected by cardioprotection elicited by administration of IPC (Fig. 6Aii: AAR/LV% Histology 58.0 ± 8.4, ASL 53.9 ± 14.3, paired t-test p = 0.33). Bland Altman analysis suggested a small bias of −4.1% (Fig. 6Bii).

ASL perfusion mapping quantification of putative AAR was not affected by CsA treatment: CsA treatment significantly increased the mean perfusion in the area considered to be normal (Supp. Table 3, mean ‘normal’ perfusion from 1 SD threshold [mg/g/ml]: vehicle group 16.9 ± 0.8, n = 6 versus CsA group 28.9 ± 11.1, n = 8, one-way ANOVA with Bonferroni test p = 0.02). There was no significant difference in the mean perfusion value in areas deemed to have ‘reduced’ perfusion by the 1 SD threshold (Supp. Table 3). However, despite the effect of CsA on increasing myocardial salvage, cardioprotection by CsA treatment did not affect quantification of putative myocardial IS and AAR by ASL perfusion mapping (Fig. 5Aiv: IS/LV% Histology 20.0 ± 4.7, ASL 23.6 ± 20.3, paired t-test p = 0.69 and Fig. 6Aiv: AAR/LV% Histology 55.3 ± 12.1, ASL 61.5 ± 12.6, paired t-test p = 0.1). Bland Altman analysis provided bias of 3.6% and 4.3% for 2 SD and 1 SD thresholds, respectively (Figs 5Biv and 6Biv).

### ASL can be used to assess myocardial physiology in studies of cardioprotection

**Figure 7:**
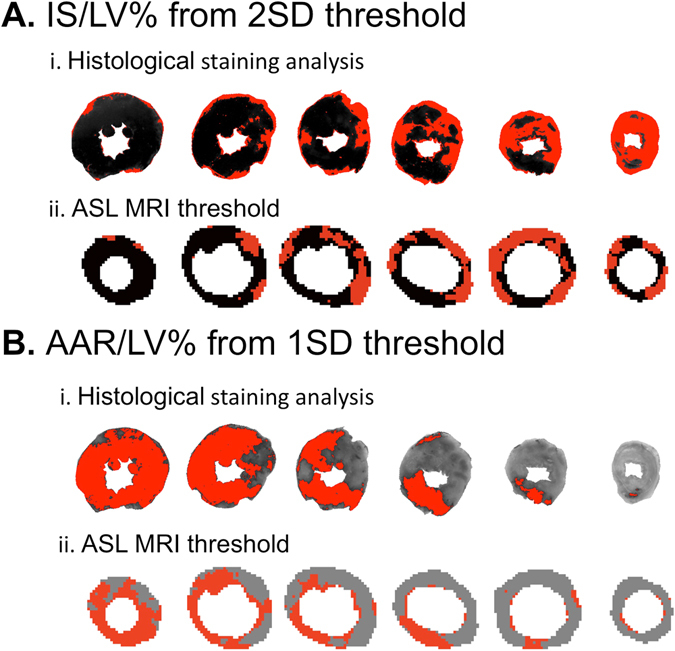
Comparison of histological staining regional analysis 1 SD and 2 SD thresholds for ASL perfusion mapping. (**A)** Quantification of IS/LV% from TTC histological staining using ImageJ planimetry where red signal represents infarcted tissue (i). ASL perfusion mapping analyzed with 2 SD threshold for IS/LV% with red signal representing putative infarct region (ii). Histology and MR images have different slice thicknesses and cardiac contraction states and therefore should not be compared slice-by-slice. (**B)** Quantification of AAR/LV% from histological staining using ImageJ planimetry where grey signal representing putative AAR region and red signal indicating normal Evans blue perfusion (i). ASL perfusion mapping analyzed with 1 SD threshold for AAR/LV% with grey signal representing putative AAR region and red signal indicating normal perfusion (ii).

**Figure 8:**
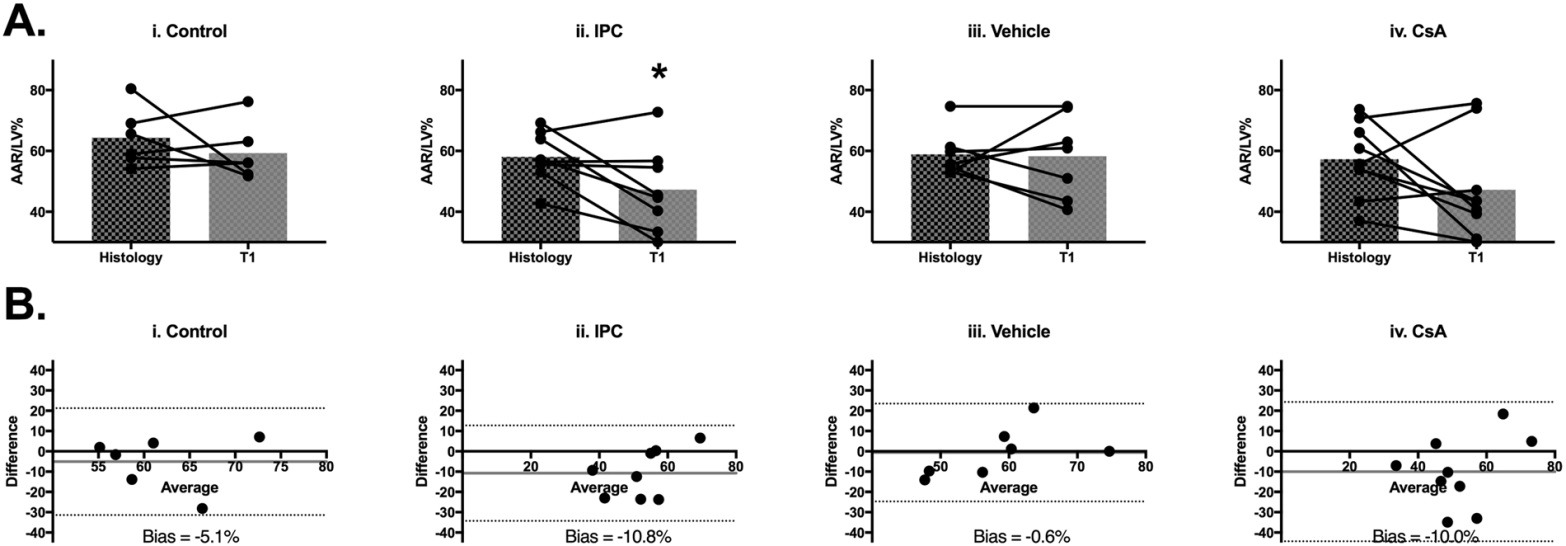
T_1_ mapping for *in vivo* AAR to determine influence on the ASL signal. (**A**) Quantification of AAR as a fraction of left ventricle area (AAR/LV%) by histological staining and T_1_-mapping in animals in control group (n = 6) (i), IPC group (n = 10) (ii), vehicle group (n = 8) (iii) and CsA group (n = 9) (iv). Mean ± standard deviation values are provided in Supp. Table 2. Significant underestimation of AAR/LV% is observed for the IPC group (*p = 0.04). (**B)** Bland-Altman plots comparing histological and T_1_-mapping methods of estimating AAR/LV% for all groups (i–iv).

Overall, there appears to be more variability using the 2 SD threshold to calculate IS/LV% compared to the 1 SD threshold to calculate AAR/LV% (Figs 7 and 8). Since neither of the cardioprotective interventions tested in this study had any significant effect on quantification of myocardial AAR by *in vivo* ASL perfusion mapping, this imaging technique is promising to quantify myocardial salvage in these animals.

### Putative AAR from T_1_ mapping

Since ASL relies on T_1_ mapping for perfusion quantification, putative AAR from T_1_ mapping was determined in order to investigate the possibility that putative AAR from ASL perfusion mapping is affected by T_1_ measurement of edema.

Quantification of the putative AAR/LV% by T_1_-mapping showed good agreement between T_1_-mapping and histological staining for control animals (Fig. 8Ai: AAR/LV% Histology 64.3 ± 9.6, T1-mapping 59.3 ± 9.2, n = 6, paired t-test p = 0.4) and CsA animals (Fig. 8Aiv: AAR/LV% Histology 57.3 ± 12.1, T1-mapping 47.2 ± 16.6, n = 9, paired t-test p = 0.12). Bland-Altman bias was −5.1% and −10.0% for control and CsA groups (Fig. 8Bi and iv).

However, underestimation of putative AAR by T_1_-mapping was observed for IPC animals due to reduced edema (Fig. 8Aii: AAR/LV% Histology 58.0 ± 8.4, T1-mapping 47.3 ± 13.8, n = 8, paired t-test p = 0.04) with Bland-Altman bias −10.8% (Fig. 8Bii). As previously described, the ASL quantification of putative AAR is unaffected by reduced edema in IPC animals, and suggesting the ASL quantification of AAR is independent from T_1_-based edema measurements.

## Discussion

The major findings of the current study are as follows: (1) T_2_-mapping performed 3 days following an AMI can accurately quantify the AAR in the murine heart in the absence of a cardioprotective intervention; (2) The cardioprotective intervention, IPC, but not CsA, reduced the extent of myocardial edema on T_2_ maps, leading to an underestimate of the AAR in IPC-treated hearts; (3) ASL perfusion mapping performed 3 days following an acute MI showed reduced perfusion outside the MI zone and could also accurately quantify the AAR in the murine heart in the absence of a cardioprotective intervention; and (4) Unlike T_2_-mapping, the AAR measured by ASL perfusion mapping was not affected by either IPC or CsA, suggesting that ASL perfusion mapping may be a more robust method for the *in vivo* quantification of the AAR in cardioprotection studies.

The reliable and accurate measurement of the AAR is required to assess myocardial salvage in clinical studies investigating novel cardioprotective interventions in acute MI patients. The current ‘gold-standard’ for achieving this is by myocardial SPECT imaging but this technique has a number of disadvantages including a substantial ionizing radiation exposure, requires round the clock access to a radioactive isotope and it suffers with poor resolution^3^.

In its place, T1 and T_2_-mapping has emerged as a technique for retrospectively quantifying the AAR in acute MI patients^19^. This has been validated against AAR measured by classical histological methods in animal AMI models^10–13^, and in patients it has been compared to AAR determined by angiography jeopardy scores^14^ and myocardial SPECT imaging^15^. However, T_2_-mapping is an indirect method for delineating the AAR, which relies on the detection of myocardial edema within the AAR. Although it is known that myocardial edema develops in response to acute IRI, the underlying pathophysiology and its time-course in the reperfused heart remain unclear^9^. Furthermore, recent clinical studies have demonstrated that certain cardioprotective interventions may reduce the extent of myocardial edema delineated by T_2_-mapping resulting in an underestimate of the AAR by this method. Thuny *et al*.^18^ found that in those STEMI patients randomized to receive ischemic postconditioning (IPost) (interrupted myocardial reperfusion following primary percutaneous coronary intervention), the extent of myocardial edema imaged by T_2_-mapping was reduced by 32%. Reductions in myocardial edema imaged by T_2_-weighted and T_2_-mapping have also been reported in clinical studies investigating the cardioprotective intervention remote ischemic conditioning (RIC) (cardioprotection induced by transient limb ischemia)^5, 6, 8^. In our study, we show for the first time that the cardioprotective intervention, IPC, reduced the extent of myocardial edema imaged by T_2_-mapping in a murine AMI model, consistent with the notion that ‘ischemic conditioning’ reduces myocardial edema as well as limiting MI size.

Interestingly, CsA did not affect the area of myocardial edema and T_2_-mapping provided a reasonably accurate quantification of AAR in the murine AMI model. This suggests that despite the cardioprotective effect of CsA in reducing MI size, it does not appear to affect the development of myocardial edema. This could perhaps be taken as evidence that these interventions exert differential protective effects against acute IRI. The potential differential effects of IPC and CsA on the extent of myocardial edema could provide support for the previous clinical studies which showed that the mechanical interventions, IPost and RIC, but not the pharmacological intervention metoprolol^6^ or exenatide^7^ reduced myocardial edema on T_2_-mapping. However, these early studies do not provide sufficient evidence of a mechanistic difference between these mechanical and pharmacological interventions for which extensive further studies are now required and are beyond the scope of this proof-of-concept study.

Given the problems associated with using T_2_-mapping to delineate the AAR, we performed a multi-parametric CMR study to investigate *in vivo* quantification of the AAR in the reperfused heart. In addition to standard LGE and T_2_ mapping, this study examined the use of a contrast-free ASL perfusion mapping approach to evaluate tissue perfusion in reperfused mouse hearts. In humans, perfusion-based cardiac CMR has mainly examined the use of first-pass perfusion imaging where the perfusion of a contrast agent is used to identify areas of differential perfusion. The success of contrast perfusion methods in the mouse heart has been limited by the spatial and temporal resolution due to its small size and rapid heart rate. The multi-slice ASL sequence used in this study has been extensively optimized for *in vivo* imaging of the mouse myocardium^20, 21^. The studies undertaken in here represent the first application of a cardiac ASL sequence for the multi-slice assessment of acute MI.

ASL perfusion maps of the reperfused myocardium showed spatially distinct regions of reduced tissue perfusion. Given that the total area of perfusion deficit was always larger than the area of infarction, it is unlikely that the measurement here reflects decreased perfusion within the infarcted area only, although perfusion is further reduced in infarcted tissue. One previous study^22^ used single slice ASL for myocardial perfusion assessment following an IRI model with 1 hour of occlusion and visually compared perfusion deficit to area of infarction (IS). Using a single slice acquisition, a “normal perfusion” region is not available for calibration, therefore it is not possible to assess the percentage of tissue exhibiting deficit to determine if this is AAR or infarct region. In our study, quantification of the area of myocardium exhibiting decreased myocardial perfusion (according to a 1 standard deviation threshold) displayed good agreement with the AAR calculated by histological staining for all groups. Quantification of IS from ASL perfusion mapping (according to a 2 standard deviation threshold) was also possible, but was subject to substantially increased variability within groups. The T_1_ maps used to generate ASL perfusion measurements also showed underestimation of AAR in the IPC group, indicating that putative AAR measured by ASL perfusion mapping is not simply mirroring T_1_ measurements of edema. Potentially confounding factors such as partial volume, resolution limits and T_1_ and T_2_ contributions to ASL signal will be investigated in more detail in future studies. This study provides the first suggestion that multi-slice ASL perfusion mapping may provide a means to determine myocardial AAR in reperfused hearts.

The reduced perfusion within the AAR is not expected to correspond to unsuccessful reperfusion of the LAD coronary artery in this model but most likely represent some impairment of microvascular perfusion within the capillary bed of these hearts. The biological basis of the perfusion deficit identified within the myocardial AAR has not been investigated here and is beyond the scope of this proof-of-concept study. It is worth noting that the ASL perfusion mapping generated more variability than other metrics in the IPC group. This study showed that neither IPC nor CsA significantly affected the quantification of AAR by ASL perfusion mapping, thereby showing promise as a more robust CMR technique for delineating the AAR. Further studies are required to compare these perfusion measurements with SPECT or microsphere perfusion measurements and validate these findings in IRI models.

Interestingly, we found that there was no significant effects of the cardioprotective treatments IPC and CsA on cardiac volumes and function, although there was a small reduction in LV mass with CsA. There may have been no effects of IPC and CsA on cardiac volumes as this was assess at 3 days post-MI and this may have been too soon to have seen any beneficial effects on LV remodeling. The failure to observe any significant improvement in cardiac function with IPC and CsA may be explained, in part, by the variability in cardiac function, the effect of myocardial stunning (reversible cardiac dysfunction), and the relatively small sample sizes.

### Limitations

It is possible that other cardioprotective interventions may affect the validity of AAR quantification by ASL perfusion mapping. It may be expected that if an intervention were to modify myocardial perfusion that this may in turn affect the calculation of AAR by ASL perfusion mapping, since this method is based on detection of a perfusion deficit. Indeed, nitrate treatment has been shown to improve myocardial perfusion in reperfused canine hearts. Interestingly, analysis of myocardial perfusion by ASL showed that CsA treatment significantly increased the rate of ‘normal’ perfusion in the non-AAR. Since an equivalent increase was not observed in IPC treated animals, it is unlikely that this corresponds to increased cardiac function in these animals. Although this remains an intriguing and unexplained effect of CsA, it did not influence the calculation of AAR by this method since the area of decreased perfusion was defined by a threshold approach based on this normal value. This study therefore suggests that ASL perfusion mapping may provide a valid method for AAR quantification even in the presence of cardioprotective interventions that increase myocardial perfusion. This should be investigated further in order to fully validate the use of this MR method for assessing myocardial salvage. In this study pixel-wise comparison between different CMR methods was not possible because of the differing cardiac phase used during imaging. Furthermore, slice-wise comparison with histological staining was not possible because of different slices thickness (created by hand slicing of frozen *ex vivo* hearts) and different contractile states.

In conclusion, this study provides a multi-parametric assessment of IS and AAR *in vivo* following IRI. ASL perfusion mapping appears to be a promising alternative method for quantifying the AAR, which unlike T_2_-mapping and T_1_-mapping, is not affected by IPC. Further studies are now required to complete the validation of ASL perfusion mapping for AAR quantification to support its potential wider future use.

## Methods

All animal experiments and study protocols were approved by The Hatter Cardiovascular Institute authorities (University College London - license number PPL 70/7140 and PPL 70/8556) and were conducted in accordance with the Animals (Scientific Procedures) Act 1986 published by the UK Home Office and the Guide for the Care and Use of Laboratory Animals published by the US National Institutes of Health 1996. B6/SV129 male mice were purchased from Harlan Laboratories, UK and were aged 10–14 weeks at the time of surgery. All laboratory reagents were purchased from Sigma, UK unless otherwise stated.

### Acute myocardial infarction *in vivo*

The established *in vivo* experimentation was exactly carried out as previously described^23, 24^. Briefly, Mice were anesthetized by inhalation of isoflurane (Isoflo, Abbott Animal Health, USA) vaporized in oxygen (1.5–1.8% isoflurane in 1.5 L/minute oxygen). Body temperature was maintained at 36.5 ± 0.5 °C. Animals were artificially ventilated (MiniVent Type 845, Hugo Sachs Electronik, Germany) and open-chest surgery performed to induce IRI. Myocardial ischemia was induced by ligation of the left anterior descending (LAD) coronary artery using an 8–0 non-absorbable polypropylene suture (Ethicon, USA) and a custom-made snare system. Myocardial reperfusion was induced by release of the occluding snare. Short lengths of the ligating suture were left *in situ* for subsequent *ex vivo* histological staining to delineate the AAR by Evans blue staining.

Mice were recovered and analgesia was provided by buprenorphine (Vetergesic, Alstoe Animal Health, UK) 0.1 mg/kg intramuscular administered 30 minutes prior to the completion of surgery and at 6 and 24 hours post-surgery.

#### Treatment groups

Mice were randomized to control or intervention groups for each study. Treatment protocols are summarized in Supp. Figure 1.

*IPC study*. control mice (n = 6) underwent 15 minutes stabilization, 30 minutes ischemia and 72 hours reperfusion (Supp. Fig. 1Ai); IPC treated mice (n = 10) underwent 5 minutes stabilization, one cycle of IPC of 5 minutes ischemia and 5 minutes reperfusion, followed by 30 minutes index ischemia and reperfusion (Supp. Fig. 1Aii).

*CsA study*. Control (n = 7) and CsA treated (n = 9) mice underwent 15 minutes stabilization, 30 minutes ischemia and 72 hours reperfusion (Supp. Fig. 1B). Mice were administered either vehicle (cremophor/ethanol-94%) or CsA (10 mg/kg) as a single intravenous bolus via the tail vein 5 minutes prior to the onset of reperfusion.

*Multi-parametric CMR acquisition and analysis*. Following 72 hours reperfusion, mice underwent cardiac CMR for assessment of MI size and putative AAR using LGE, T_2_-mapping and ASL perfusion mapping protocols.

*CMR setup*. Mice were anesthetized by inhalation of isoflurane vaporized in oxygen (maintained at 1.5% isoflurane in 1 L/minute oxygen). Real-time monitoring was performed of respiration (neonatal apnea sensor placed on the abdomen), ECG (two-lead setup, SA Instruments, USA) and body temperature (rectal thermometer attached to SA Instruments, USA). Body temperature was maintained at 36.5 ± 0.5 °C. CMR was undertaken using a 9.4 T horizontal bore scanner (Agilent Technologies, USA) fitted with 1000 mT/m gradient inserts (inner diameter of 60mm; Agilent Technologies, USA) running VNMRJ software (version 2.3 A). Volume resonator quadrature radiofrequency (RF) coils (RAPID biomed, Ripmar, Germany) were used for RF transmission and signal reception.

Respiration gating was achieved using respiratory bellows (described above) to trigger image acquisition immediately following exhalation. Cardiac gating was achieved by detection of the integrated ECG trace to allow triggering of image acquisition following detection of the ECG R-wave. Respiration and cardiac gating parameters are described for each CMR sequence protocol below.

CMR image analysis was performed blinded and randomized using custom scripts (MATLAB Student Version R2008b, The MathWorks, USA). This consisted of myocardial segmentation, selection of normal region of interest (ROI) and threshold analysis, as described to follow.

#### CINE MRI

Cine imaging was acquired in the short axis view using a stack of 10 slices to assess cardiac function (cardiac and respiratory gated gradient echo sequence, TE/TR = 1.2/4.5–5 ms, flip angle = 15**°**, cine frames = 20, matrix size = 128 × 128, in-plane resolution = 200 µm, slice thickness = 1 mm, 10 short-axis slices). Endomycardial segmentation was performed using the freely available software Segment (Medviso, Lund, Sweden).

#### LGE CMR

LGE CMR was conducted as described previously by Price *et al*.^25^. In brief, gadolinium diethylene-triamine penta-acetic acid (Gd-DTPA) was administered via an intraperitoneal infusion line as a single bolus of 0.6 mmol/kg Gd-DTPA (Magnevist, Germany). Following a delay of 10 minutes to allow for enhancement by Gd within the area of infarction, a set of T_1_-weighted images was acquired. A Look-Locker-style gradient echo acquisition was used to determine the null point of healthy myocardium in one mid-ventricle slice (one acquisition per cardiac cycle, TR = RR-interval, TE = 1.1 ms, TI range ~ 100–900 ms, field-of-view (FOV) = 25.6 mm × 25.6 mm, slice thickness = 1 mm, flip angle = 10°, recovery delay = 1 s, acquisition time ~ 3 minutes). LGE images were acquired using the single inversion time point chosen with Look-Locker acquisition (typically 3 RR-intervals) (7 short axis slices acquired sequentially during systole per cardiac cycle, TE = 1.1 ms, TR = 3.1 ms, FOV = 25.6 mm × 25.6 mm, matrix = 128 × 128, slice thickness = 1 mm, flip angle = 10°).

### Multi-parametric CMR acquisition

The area of infarction was determined using an automated delineation method constrained by the myocardial borders using Segment software (version 8, Medviso, Sweden). Infarct size was calculated as the number of pixels with gadolinium enhanced T_1_-signal as a percentage of the total number of pixels.

#### T_2_-mapping

T_2_ mapping was performed using a cardiac and respiration gated spin-echo sequence, with sequence parameters as follows: TE = (3.5, 7, 10, 12, 15, 17, 20, 25, 30) ms, TR = RR-interval, matrix = 128 × 128, FOV = 25.6 mm × 25.6 mm, 7 short axis slices (one per cardiac cycle), slice thickness = 1 mm. A delay following the R-wave was introduced to ensure that the MR signal was measured at the same cardiac phase.

Exponential decay curves of T_2_-relaxation time were fit to the myocardial signal intensity on a pixel-wise basis for each slice using MATLAB scripts written in-house. Normal T_2_ map signal values were defined by selection of an ROI in the second from most basal slice, which is expected to be normal (corresponding to non-AAR) in this mouse model of IRI. Pixels with T_2_ values greater than 1 standard deviation above the ‘normal’ T_2_ were considered to have elevated T_2_ values^26^. The area of elevated T_2_-signal was calculated as a percentage of the total number of myocardial pixels (area of elevated T_2_/myocardial area %).

#### ASL perfusion mapping

Myocardial perfusion was quantified using a multi-slice flow alternating inversion recovery (FAIR) ASL sequence^20, 21^. T_1_-relaxation was measured following slice-selective (control condition) and global (tagged condition) inversion RF pulses using a multi-slice segmented ECG-gated Look-Locker method. Scanning parameters were as follows: TE/TR(inv)/TR(RF) = 1.18 ms/13.5 s/3 ms, flip angle = 5°, FOV = 25.6 mm × 25.6 mm, matrix = 128 × 128, slice thickness = 1 mm, 6 slices total (3 slices/acquisition), number of points in recovery curve = 50. The inversion pulse was respiratory and cardiac gated, and the Look-Locker acquisition was only cardiac gated.

Multi-slice perfusion maps were calculated by comparison of T_1_-recovery curves following slice-selective and global inversion pulses, incorporating a blood pool input function (bpMBF quantification)^19^. This was performed on a pixel-by-pixel basis to generate ASL perfusion maps for each heart slice using MATLAB scripts written in-house. Normal perfusion values were defined for each heart by selection of a ROI in the second from most basal slice. Two thresholds were compared to assess perfusion deficit: pixels with perfusion values less than 1 standard deviation (1 SD) and 2 standard deviations (2 SD) below the ‘normal’ perfusion value for that heart. The area of reduced perfusion was calculated as a percentage of the total number of myocardial pixels (area of perfusion deficit/myocardial area %) for each threshold.

#### T_1_-mapping

The T_1_ maps used for ASL perfusion quantification (following global inversion pulse) were also analyzed for areas of increased T_1_ corresponding to edema. Again, normal T_1_ was defined by the ROI in the second from most basal slice and T_1_ values great than 1 standard deviation above normal were considered elevated T_1_.

#### Histological staining to quantify MI size and AAR

In order to validate cardiac CMR measurements of infarct size and the AAR, hearts were subjected to *ex vivo* histological staining using triphenyl-tetrazolium chloride (TTC) and Evans blue for determining MI size and AAR, respectively. Briefly, immediately following completion of CMR, mice were sacrificed by administration of ketamine 10 mg/ml (Vetalar, Boehringer Ingelheim, UK), xylazine 2 mg/ml (Rompun, Bayer, UK) and atropine 0.06 mg/ml (Sigma, UK) at 0.2 ml/10 g. Hearts were rapidly extracted and the aorta cannulated to allow retrograde perfusion. Residual blood was removed by perfusion of saline (pre-warmed to 37 °C). MI size was delineated by perfusion of TTC (7 ml of 1% TTC in phosphate-buffered saline [PBS], pre-warmed to 37 °C). The occluding ligature used to induce ischemia was securely re-occluded and Evans blue dye (1.5 ml 0.5% Evans blue in distilled water) perfused to delineate the AAR. Hearts were stored at −20 °C to facilitate manual slicing of 7 transverse slices of approximately 1 mm thickness from the apex to the base of the left ventricle. Myocardial slices were briefly incubated in 10% formalin at room temperature and imaged (Epson Perfection V100 Photo, Epson, UK) in a custom-made acrylic block.

Quantifications were performed on transverse slices of the left ventricle using ImageJ planimetry (NIH, USA) and infarct size (IS) and AAR were expressed as a fraction of LV area (IS/LV% and AAR/LV%). Infarct size as fraction of AAR was also calculated (IS/AAR%).

### Statistical analysis

All statistical analysis was completed using GraphPad Prism® version 7.0 (GraphPad Software, USA). Differences were considered significant where p < 0.05; p-values are provided alongside for statistical analysis.

#### Areas

Percentage areas are reported for the left ventricle (LV) as mean ± standard deviation. Comparison of area quantifications between groups was by unpaired t-test where two groups were compared and by one-way ANOVA and Bonferroni test where more than two groups were compared. Comparison of area quantifications within groups was by paired t-tests. Bland-Altman plots were generated to compare histological and CMR parameters for individual animals. Biases from Bland-Altman analyses are reported.

#### Quantitative T_2_, perfusion and T_1_ values

Mean T_2_ (ms) ASL perfusion (ml/g/min) and T_1_ (s) values were calculated for areas deemed ‘normal’ and ‘elevated’ or ‘reduced’ by threshold analysis. Mean values were compared for each study using one-way ANOVA and Bonferroni test where results are reported as multiplicity adjusted p-values.

## Electronic supplementary material

Supplementary information
