# Supplementary material for: Quantifying the area-at-risk of myocardial infarction in-vivo using arterial spin labeling cardiac magnetic resonance
Source: Sci Rep. 2017 May 23;7:2271. doi: 10.1038/s41598-017-02544-z (PMC5442118; doi:10.1038/s41598-017-02544-z)

Supplementary Information

Quantifying the area-at-risk of myocardial infarction *in-vivo* using arterial spin labeling cardiac magnetic resonance

Rachel K Dongworth*[1], Adrienne E Campbell-Washburn*[2,3],
Hector A Cabrera-Fuentes[4,5,6,7,8], Heerajnarain Bulluck MD[1], Thomas Roberts[2], Anthony N Price[9], Sauri Hernández-Reséndiz[4,5], Roger J Ordidge[10], David L Thomas[11], Derek M Yellon[1,12], Mark F Lythgoe[2], Derek J Hausenloy[1,4,5,12,13,14]

*Joint first authors

[1] The Hatter Cardiovascular Institute, University College London, UK
[2] Centre for Advanced Biomedical Imaging, University College London, UK.
[3] Biochemistry and Biophysics Center, Division of Intramural Research, National Heart, Lung and Blood Institute, National Institutes of Health, Bethesda, USA.
[4] Cardiovascular and Metabolic Disorders Program, Duke-NUS Graduate Medical School, Singapore, Singapore
[5] National Heart Research Institute Singapore, National Heart Centre Singapore, Singapore, Singapore
[6] Department of Cardiovascular Biomedicine, National Institute of Cardiology I. Ch, Mexico, Mexico City
[7] Institute of Biochemistry, Medical School, Justus-Liebig-University, Giessen, Germany;
[8] Kazan Federal University, Department of Microbiology, Kazan, Russian Federation;
[9] Division of Imaging Sciences and Biomedical Engineering, King's College London, King's Health Partners, St. Thomas' Hospital, London, UK
[10] Centre for Neuroscience, University of Melbourne, Melbourne, Australia
[11] Department of Brain Repair and Rehabilitation, UCL Institute of Neurology, University College London, London, United Kingdom; Leonard Wolfson Experimental Neurology Centre, UCL Institute of Neurology, University College London, London, United Kingdom
[12] The National Institute of Health Research University College London Hospitals Biomedical Research Centre, London, UK
[13] Yong Loo Lin School of Medicine, National University Singapore, Singapore
[14] Barts Heart Centre, St Bartholomew's Hospital, London, UK.

SUPPLEMENTARY TABLES

Supplementary Table 1

Table 1: IS/LV% values

IS/LV% from histological staining compared to LGE and ASL (2SD threshold). The number of histological slices used for analysis matches the spatial extent of each CMR method

	Histology (7 slices)			T2-mapping				Histology (6 slices)			ASL perfusion mapping				T1-mapping			
Control	64.3	±	6.1	60.3	±	5.8	(p = 0.43)	64.3	±	9.6	65.9	±	5.6	(p = 0.71)	59.3	±	9.2	(p = 0.40)
IPC	59.8	±	7.9	50.7	±	5.4	(p = 0.02)	59.8	±	7.9	53.9	±	14.3	(p = 0.33)	47.3	±	13.8	(p = 0.04)
Vehicle	65.4	±	7.0	65.4	±	10.1	(p = 0.32)	61.4	±	7.0	57.9	±	5.0	(p = 0.79)	58.3	±	13.8	(p = 0.90)
CsA	58.0	±	12.6	62.8	±	13.1	(p = 0.32)	57.3	±	12.1	61.5	±	12.6	(p = 0.10)	47.2	±	16.6	(p = 0.12)

Supplementary Table 2

Table 2: AAR/LV% values

AAR/LV% from histological staining compared to T_2 mapping, ASL (1SD threshold) and T_1-mapping. The number of histological slices used for analysis matches the spatial extent of each CMR methods.

	Histology (7 slices)	LGE		Histology (6 slices)	ASL perfusion mapping	
Control	32.3 ± 3.7	31.7 ± 5.8	(p = 0.74)	31.5 ± 4.8	24.8 ± 11.2	(p = 0.28)
IPC	16.8 ± 3.0	17.8 ± 4.9	(p = 0.77)	15.6 ± 3.3	26.6 ± 17.9	(p = 0.45)
Vehicle	34.9 ± 9.6	29.2 ± 5.8	(p = 0.07)	29.0 ± 8.3	25.1 ± 11.8	(p = 0.14)
CsA	21.9 ± 4.5	19.2 ± 6.9	(p = 0.44)	20.0 ± 4.7	23.6 ± 20.3	(p = 0.69)

	Histology (7 slices)	T_2 -mapping		Histology (6 slices)	ASL perfusion mapping		T_1 -mapping	
Control	64.3 ± 6.1	60.3 ± 5.8	(p = 0.43)	64.3 ± 9.6	65.9 ± 5.6	(p = 0.71)	59.3 ± 9.2	(p = 0.40)
IPC	59.8 ± 7.9	50.7 ± 5.4	(p = 0.02)	59.8 ± 7.9	53.9 ± 14.3	(p = 0.33)	47.3 ± 13.8	(p = 0.04)
Vehicle	65.4 ± 7.0	65.4 ± 10.1	(p = 0.32)	61.4 ± 7.0	57.9 ± 5.0	(p = 0.79)	58.3 ± 13.8	(p = 0.90)
CsA	58.0 ± 12.6	62.8 ± 13.1	(p = 0.32)	57.3 ± 12.1	61.5 ± 12.6	(p = 0.10)	47.2 ± 16.6	(p = 0.12)

Supplementary Table 3

Table 3: Quantitative values from T_2 mapping, perfusion mapping and T_1 mapping

Quantitative normal and elevated or reduced values for T_2 mapping, perfusion mapping (1SD and 2SD thresholds) and T_1 mapping. p values from one-way ANOVA with Bonferroni correction are given to compare Control vs. IPC groups and Vehicle vs. CsA groups.

	**T2 [ms]**		**Perfusion (1std threshold) [ml/g/min]**	
	Normal	Elevated	Normal (1std)	Reduced (1std)
Control	17.8 ± 2.3	21.5 ± 3.0	14.0 ± 3.4	4.3 ± 1.0
IPC	19.6 ± 3.4 (p > 0.9999)	26.8 ± 5.4 (p = 0.07)	21.0 ± 5.0 (p = 0.47)	5.4 ± 2.8 (p > 0.9999)
Vehicle	17.3 ± 1.9	22.7 ± 2.4	17.0 ± 0.8	1.6 ± 1.6
CsA	17.7 ± 1.9 (p > 0.9999)	23.8 ± 2.5 (p = 0.95)	28.9 ± 11.1 (p = 0.02)	7.8 ± 4.4 (p = 0.41)

	**Perfusion (2 SD threshold) [ml/g/min]**		**T1 [s]**	
	Normal (2std)	Reduced (2std)	Normal	Elevated
Control	8.2 ± 2.3	2.3 ± 0.9	1.7 ± 0.1	1.9 ± 0.1
IPC	13.7 ± 6.1 (p = 0.47)	0.6 ± 3.6 (p > 0.9999)	1.7 ± 0.3 (p > 0.9999)	2.0 ± 0.3 (p > 0.9999)
Vehicle	12.7 ± 1.6	1.2 ± 3.3	1.5 ± 0.5	2.0 ± 0.1
CsA	19.8 ± 7.6 (p = 0.13)	2.3 ± 4.4 (p > 0.9999)	1.6 ± 0.4 (p > 0.9999)	2.0 ± 0.1 (p > 0.9999)

SUPPLEMENTARY FIGURES

Supplementary Figure 1: Treatment groups for murine *in vivo* IRI

Mice were randomized to control or treatment groups for each study.

A. IPC study: control group received standard IRI protocol of 15 minutes stabilization, 30 minutes index ischemia and 72 hours reperfusion. IPC group received 5 minutes stabilization, one cycle of IPC consisting of 5 minutes ischemia and 5 minutes reperfusion, followed by 30 minutes index ischemia and 72 hours reperfusion.

B. CsA study: both groups received standard IRI protocol of 15 minutes stabilization, 30 minutes index ischemia and 72 hours reperfusion. Control group received a matched volume dose of vehicle (cremophor / ethanol-94%). CsA group received CsA 10mg/kg. Vehicle and CsA were administered as single intravenous dose delivered 5 minutes prior to the onset of reperfusion.

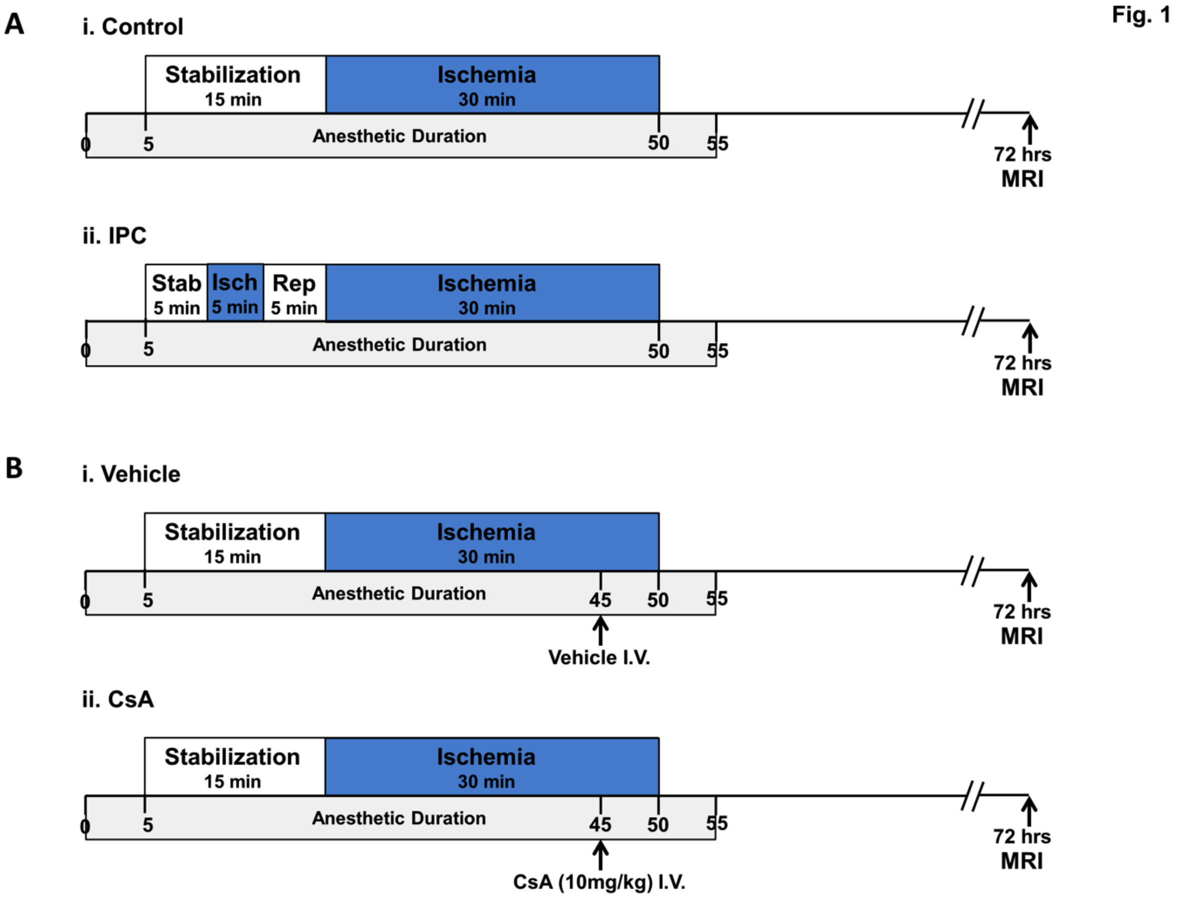

Supplement: Supplementary file 1 — Supplementary information [file 41598_2017_2544_MOESM1_ESM.pdf]
